# The Rurality of Intimate Partner Femicide: Examining Risk Factors in Queensland

**DOI:** 10.1177/10778012231158105

**Published:** 2023-02-22

**Authors:** Freya McLachlan

**Affiliations:** 1 1969 Queensland University of Technology, Brisbane, Queensland, Australia

**Keywords:** rural, intimate partner femicide, isolation

## Abstract

While violence against women and domestic violence can be seen throughout Australia, emerging evidence suggests that intimate partner femicide (IPF) is more common in rural spaces than urban ones. This study examined 100 IPF cases to determine the rate of femicide and frequency of common risk factors in rural areas of Queensland, Australia. The study also explored how victims accessed services and the characteristics of rural IPF and male offenders. Findings indicated that IPF is more common in rural areas and associated risk factors are similar between urban and rural cases. Rural IPF was more likely to occur during a current relationship and offenders were found to be less likely to conceal their actions. These differences suggest that the physical and social isolation of rural spaces may facilitate higher rates IPF. Implications discuss the need for rural-focused policies and responses.

## Introduction

Within a year in Australia, a woman is killed approximately every 9 days by an intimate partner (Australian Institute of Health and Welfare [AIHW], 2019). The AIHW (2019) estimates that at least one in six women and one in 16 men have experienced some form of intimate partner violence (IPV) since the age of 15. This rate of violence for women increases further when environment is considered, with 21% of women living outside of a major city experiencing IPV since the age of 15, compared to 15% of those living in a major city (Australian Bureau of Statistics, 2013). It must be noted however that this figure is likely much higher, particularly in regional and remote areas where domestic violence (DV) is significantly underreported due to traditional gender norms and a normalization of male violence and control (George & Harris, 2014). Although rates of DV and IPV are higher in rural areas when compared to urban spaces, there is a dearth of literature that examines the impact that these rates have on the risk factors and lethality of this violence.

DV encompasses a range of physical and nonphysical abuse that occurs within family relationships or between intimate partners (Dawson & Piscitelli, 2017). DV and abuse includes but is not limited to physical assault, sexual assault, stalking, economical abuse, threatening behavior, and psychological and emotional abuse (Queensland Government, 2019). Coercive and controlling behaviors also fall under this definition, encapsulating widely impactful abuse that is often difficult to identify (Stark, 2007). Internationally, a variety of definitions have been applied to the phenomenon of DV based on classifications of violence and domestic relationships (Fairbairn et al., 2017). This research will use the term DV to refer to the broader abuse within any domestic relationship, and IPV for the violence between current or previous intimate partners (McCulloch et al., 2016; McEwan et al., 2017). While DV is more prevalent in certain cohorts in Australia such as Indigenous communities, it is widespread across all cultures, genders, communities, and ages (Bugeja et al., 2013). Intimate partner homicide, or more specifically intimate partner femicide (IPF), is the murder of a woman perpetrated by a current or former intimate partner. While intimate partner homicide more broadly, which includes male and female victims, can and does occur, the overwhelming majority of victims are female, while perpetrators are largely male. In light of this, while acknowledging Stark (2007) and Johnson's (2008) frameworks of gendered violence, IPF is studied in this paper, firstly as the scope of the research and secondly to highlight the nature of this violence within a patriarchal and gendered society.

Compared to other western countries such as the United States, the rate of homicide in Australia is extremely low, though this does not lessen the impact of this violence (McKinley, 2017). Domestic homicide, the murder of a person by a family member or intimate partner, accounts for the largest portion of homicides in Australia (Bryant & Bricknell, 2017). In 2019, Queensland reported 53 homicides with 10 of these being IPF (Queensland Government, 2020; Queensland Government Statistician's Office, 2021). General and domestic homicide rates are highest in rural areas, with Northern Queensland, comprising mostly remote and very remote areas, accounting for the largest portion of homicides in Queensland, one state in Australia (Queensland Police Service, 2016, 2017).

While these numbers appear small, the trends of IPF in Australia are in line with other high-income countries, with IPV accounting for “more death, disability and illness for women aged 15 to 44 than any other preventable risk factor” (Fitz-Gibbon et al., 2018, p. 4). Stöckl et al.'s (2013) global review of intimate partner homicide found that 13.5% of homicides worldwide could be attributed to an intimate partner, while global rates of IPF accounted for nearly 40% of femicide (p. 863). This is comparative to the rate of IPF in Australia, of which intimate partners perpetrate 51% of all femicides (Johnson et al., 2019, p. 3). There are, however, challenges in the recording and collection of data concerning femicides globally, both in the reporting of the rate of femicides and in the capture of complex risk factors related to IPF, such as coercive control and nonphysical violence (Walklate et al., 2019). Many researchers agree that the recorded rate of most risk factors associated with IPF, and IPF itself is an underrepresentation of the true extent of this violence on an international scale (Bitton & Dayan, 2019; Cullen et al., 2021; Cullen et al., 2019). Additionally, the reporting of femicides raises issues with broad comparisons of data, as there are no strict international classifications of what constitutes a femicide or IPF (Bugeja et al., 2015).

As a result of these statistics and the overrepresentation of women as victims of gendered violence, there has been a large response within Australia and Queensland both in the research conducted into domestic and IPV and homicide, as well from state and federal governments in their design of policy and interventions, with the intent to support victim/survivors and prevent further deaths from occurring. These responses have largely focused on DV; however, there is a growing body of research that has begun to examine IPF in Australia (see Boxall et al., 2022; Cullen et al., 2019; Ferguson & McLachlan, 2020; Fitz-Gibbon et al., 2018; Johnson et al., 2019; McLachlan & Harris, 2022; Walklate et al., 2019). Within the last decade, scholars in Australia have begun to examine previously overlooked or unexplored risk factors of DV, IPV, and domestic homicide such as the use of technology, a lack of physical violence, and the focus of this paper—the rurality of the environment in which some homicides occur. With the implementation of the Domestic and Family Violence Death Review Unit (DFVDRU) in Queensland in 2011 following similar teams around the country, strides have been made in understanding domestic homicide. Despite extensive discussion of isolation, both geographically and socially, an emerging question in academic literature Australia wide, is how IPV and IPF offenders differ in different environments (Beyer et al., 2015).

Research has previously indicated that a large portion of domestic homicides in rural areas may begin as assaults that would not be lethal if not for the isolation of the environment (Websdale, 1999). Australia is unique in its composition of populated areas, as much of the country is considered remote or very remote (Baxter et al., 2011). Most major cities are located coastally, with regional and remote areas expanding inland. Because so much of the country is considered remote, the examination of violence in areas that are not urban centric is vital in understanding and ultimately reducing IPV and IPF across Australia (Harris, 2016). Previous research has examined the presentation of DV and IPV in rural Australia (notably Dillon et al., 2015; George & Harris, 2014; Harris, 2016; Harris & Woodlock, 2019; Owen & Carrington, 2015) and there is a consensus that this violence is frequent and perhaps more prevalent when compared to urban areas. This paper adds to this growing body of literature with the unique contribution of examining the rate and associated risk factors of IPF that occurs in regional and remote areas in Queensland, and how perpetrators differ from those in urban areas. This exploration is important not only in furthering the understanding of IPF in Queensland, but also nationally and internationally valuable in providing data for comparison. The research questions are as follows:

- What are the features of IPF in rural areas of Queensland, Australia?
- How do victims of IPF seek help before the femicide?
- Are the behaviors or risk factors for male offenders in rural areas unique?

## Literature Review

### The Country Idyll

The idea of country living, “escaping to the country,” is often based in a westernized and romanticized ideal of what living rurally looks like. Generally, there is a misconception in non-Indigenous communities that small towns or rural communities are free of crime and are safer than living in urban-centric areas (Strand & Storey, 2019). In Australia, however, crime rates in regional and remote areas are in actuality much higher than they are perceived to be (Owen & Carrington, 2015). A term conceptualized by Hogg and Carrington (2006, p. 17) “the architecture of rural life” explains that this country idyll is based in both the perception of the culture of rural life as well as the physicality of the landscape. The concept of rurality exceeds simply the geographic location and instead is based on the culture, experiences, and perspectives of an individual (Straatman et al., 2020). The popularity of country life has waxed and waned as gentrification has dominated smaller towns and those who reside rurally have moved to larger cities to pursue education, employment, and further opportunities (Cloke, 2006).

### Rates of Crime in the Country

DV is often treated as a metropolitan problem, both in its exploration and solutions. This is likely due to its visibility and relative closeness to services that can provide intervention in urban areas (Gallup-Black, 2005). The crime rates in urban areas, particularly homicides, are thought to be highest in those areas which are densely populated and often considered dangerous in comparison to idyllic country towns (Gallup-Black, 2005). When examined more closely; however, it becomes clear that violent crime, including homicides, are more common and more likely to be committed by a family member or intimate partner (Strand & Storey, 2019), and this can be seen in the rate of domestic homicides in rural Queensland (Queensland Government, 2020).

A study conducted by Carrington (2007) of crime rates in regional and remote areas of Australia found that nationally, including Queensland, violent crime rates, including DV, were highest in these rural areas when compared to urban areas. George and Harris (2014) highlight the higher levels of DV in rural areas; however, note that it is difficult to determine the true amount of this type of violence, as it is understood to be largely underreported in both urban and rural settings. While some may attribute this rate of crime to the high number of Indigenous people living outside of major cities because of the high rates of family violence in Indigenous populations, it is clear that these rates cannot be attributed to solely Indigenous communities (Owen & Carrington, 2015). Studies have consistently shown that communities made up of majority non-Indigenous persons can account for much of the reports of DV in both urban and rural locations (Campo & Tayton, 2015; Owen, 2012). International studies have found that rates of IPV are higher in rural areas over urban areas and have further found the severity and frequency of IPV to be higher in rural locations (Beyer et al., 2015; Gallup-Black, 2005; Strand & Storey, 2019).

While there is little Australian research into IPF in rural areas, some international studies have examined the rates of rural intimate partner homicide. A study from Gallup-Black (2005) found that over a 20-year period between 1980 and 1999 in the United States, rates of intimate partner homicide were higher in rural areas than in urban ones. Using 5-year averages from FBI Supplementary Homicide Report data, she observed a decrease in intimate partner homicide in urban areas while rates increased by more than 60% in rural areas (Gallup-Black, 2005, p. 159). Additionally, Gallup-Black (2005) found that those living in rural areas were more likely to be murdered by a family member or intimate partner than those in urban areas. Such findings beg the question whether Australia may experience similar trends in the expansive regional and rural areas across the country.

### Risk Factors of IPF in Rural Areas

The growing body of literature which examines risk factors to predict escalation and lethality of DV also corroborates them as being positively associated with IPF (Bridger et al., 2017; Cunha & Gonçalves, 2016; Weizmann-Henelius et al., 2012). By examining the highly rated factors in these risk assessments, researchers are also able to examine the common characteristics of IPF offenders retroactively, after a homicide has occurred. There are multiple studies that have examined these common risk factors; however, one of the most widely cited papers to highlight these characteristics is the formative review from Campbell et al. (2007). Campbell et al. (2007) found the following risk factors to be most closely associated with IPF offenders; previous IPV, stalking, pending or actual separation, ownership of lethal weapons, substance use, and mental illness. In the last decade, several other researchers have found similar associations between IPF and the same factors (see Bendlin & Sheridan, 2019; Dobash & Dobash, 2015; Vatnar et al., 2017), as well as more recently explored factors including coercive control, nonphysical violence, technology-facilitated abuse, and living rurally (Ferguson & McLachlan, 2020; Harris & Woodlock, 2019; Johnson et al., 2019; McLachlan & Harris, 2022; Woodlock, 2017).

Living rurally may be a risk of IPF because women experiencing DV in regional and remote areas face many unique barriers in seeking help and escaping DV (Owen & Carrington, 2015). The General Social Survey 2006 found that those living in regional and remote areas had more difficulty in accessing medical, employment, family, telecommunication, and financial services than those living in major cities. Research has found that rurality is a barrier for women experiencing IPV both in its geographic location and the cultural environment associated with country living (Doherty, 2017). Living rurally has therefore been examined as a risk factor for both IPV and IPF.

Research that compares the rates of IPV between urban and rural locations is rare; however, in research that has been conducted, findings highlight that rural perpetrators hold lower levels of education and employment, use alcohol and other drugs, and are more likely to have been convicted of IPV in the past (Strand & Storey, 2019). A common finding in this research is that those living in rural areas are more likely to be tolerant of IPV due to values based on patriarchal thinking, such as victim blaming and expectations of women adhering to traditional gender roles (Strand & Storey, 2019). Regional and remote areas often maintain more traditional values over urban areas and in light of this women are often expected to fulfill a role of mother and wife over individual agency (Owen & Carrington, 2015). This pressure emphasizes the stigma of abuse within family units and perpetuates the shame that leads to victim/survivors maintaining the appearance of a happy family over their own safety and wellbeing (Owen & Carrington, 2015).

The traditional values placed onto women in rural areas mean that financial stability may be difficult to sustain unless they are reliant on a man's income (Owen & Carrington, 2015). Employment opportunities are fewer for women than in urban areas and inheritance is often from man to man. Education can be interrupted or abandoned in order to fill family roles in which a woman is expected to stay at home and tend to traditionally women-centric duties such as childcare, housework, and caring for aging parents (Owen & Carrington, 2015). While these gendered values exist outside of rural areas, the added physical isolation leads to women becoming socially dependent on their male counterparts or being at risk of social isolation in rural areas more often (Harris, 2016). Those women who are culturally and linguistically diverse (CALD) or with disabilities are additionally isolated in regional and rural areas, with discrimination and a lack of access to appropriate support services contributing to experiences of violence from family and intimate partners (Campo & Tayton, 2015). There is little research examining CALD victim/survivors experiences of IPV or IPF in rural Australia, likely due to the majority of regional and remote populations being made up of white Australians and Indigenous communities (Campo & Tayton, 2015).

Additional risk factors in regional and rural areas include natural disasters such as floods and bush fires (Campo & Tayton, 2015). Research has found that DV increases following such an event and more rurally located communities often experience the worst of these disasters (Parkinson & Zara, 2013). The external stressors associated with natural disasters including loss of housing and income escalate existing abuse. The global pandemic should also be considered, as the impacts of COVID-19 reflect the loss of income and other external factors that are associated with natural disasters (Carrington et al., 2020).

There is a large amount of research that examines risk factors for violence in terms of the characteristics of the individual offender; however, a growing area of research is now examining the impact of social and societal contextual factors on rates of violence. A study conducted by Wilkins et al. (2019) examined census data and rates of violent death from 2011 in Australia, Canada, and the United States. They found that homicide and suicide were correlated with not only individual risk factors but also the social context linked with specific states in each country. Social factors such as residential instability, employment, and income inequality were associated with homicide and suicide, while gender-economic inequity was associated with suicide (Wilkins et al., 2019). Self-employment such as farming has links to living in more rural areas and in turn having access to lethal means such as firearms in Australia (Wilkins et al., 2019).

### How is Rural IPF Different to Urban IPF?

In a study of rural DV in Canada over 18 months, Doherty and Hornosty (2008) found that women living rurally were more likely to be killed by an intimate partner than those living in major cities. They further found that when compared to women in urban areas, those in rural communities were more likely to experience IPV or IPF while still living in a current relationship with the offender and alcohol and other drugs were more likely to be involved. This contradicts Australian literature that has commonly found that it is less likely for alcohol and other drugs to be involved when examining IPF generally and for risk of escalated violence to increase *after* separation (Bridger et al., 2017; Cunha & Gonçalves, 2016).

### Social Isolation

In a follow-up validation study, Doherty (2017) conducted focus groups and interviews with 27 participants in rural Canadian towns. She found that victim/survivors in rural towns were found to be more likely to disclose violence and abuse to informal community supports such as friends and family and religious leaders over governmental agencies such as police or social services due to a fear of these services that is inherent in smaller communities and more rural areas (Doherty, 2017). Victim/survivors report the impact of embarrassment and fear of being ‘outed’ or becoming the subject of gossip as a deterrent for accessing more formal DV services and identifying as a victim/survivor (Owen & Carrington, 2015). The social aspect of small communities does not act as an aid as might be expected in these cases and instead highlights the isolation of living rurally.

### Physical Isolation

Social isolation closely relates to the physical isolation that many victim/survivors experience when living in regional or remote areas. In rural locations, homes are often based on larger plots of land in which separate victim/survivors from even their neighbors, meaning that women are often reluctant or unable to escape their abuser (George & Harris, 2015). A study conducted by Strand and Storey (2019) examined how the severity of IPV is perceived and changes based on rurality and location of the violence. Their work examined risk assessments conducted between 2010 and 2014 by the Swedish police in urban, rural, and remote areas. Their results suggest that IPV risk factors are most severe in rural areas and that victim/survivors often experienced these risk factors for longer periods of time than in urban areas (Strand & Storey, 2019). This indicates that due to the isolation of rural living, IPV is more severe and can be perpetrated without intervention for longer periods of time. Without access to services and multiple barriers for victim/survivors to seek help, rural areas facilitate this violence and add to the risk of IPF (Gallup-Black, 2005). The physical distance between a victim/survivor and friends or services that might be able to provide assistance can mean that women will simply allow physical injuries to heal rather than seeking help for the physical abuse or attempting to escape their abuser (Owen & Carrington, 2015). DV services and police in rural areas often must cover vast amounts of the country, with towns and communities hundreds of kilometers apart (Owen & Carrington, 2015). This barrier of access adds to the feeling of isolation both physically and socially for victim/survivors and can lead to consistent under-reporting of DV to the police and other services, in turn increasing risk of lethal harm. Some research suggests that a large portion of the domestic homicides in rural areas likely begin as assaults that would not be lethal if health and policing services were more accessible as they are in urban areas (Websdale, 1999).

### Technology in Isolation

George and Harris (2014) examined the experiences of female survivors of DV in rural Victoria, Australia, conducting semistructured interviews with 30 survivors and 46 relevant professionals and service providers. George and Harris (2014) found that identifying and reporting DV outside of physical abuse was difficult for victim/survivors in rural areas as some services did not recognize or understand nonphysical abuse. An example of this nonphysical abuse that is commonly reported by rural DV victim/survivors is technology-facilitated abuse; however, this form of abuse has only recently begun to emerge as a valid risk factor for IPF by criminal justice agents such as police and magistrates (Douglas et al., 2019; McLachlan & Harris, 2022; Woodlock, 2017). The social and geographic isolation of the location and culture of rurality complements and enables DV as offenders can control a victim/survivor's access to technology that could be used to bridge the gap in access to services (Harris & Woodlock, 2019). A phone may be used to contact emergency services, reach out to DV and support services, or contact family and friends when physical location isolates victim/survivors (Campo & Tayton, 2015). Offenders who monitor or limit victim/survivors’ access to technology or locally based support use the rural environment as a means to abuse and control their victim (Campo & Tayton, 2015). Because of this previous lack of awareness in criminal justice agents, the reporting of technology-facilitated abuse often does not reflect the actual experiences of victims/survivors, resulting in incomplete or misleading rates of this abuse.

Existing research suggests that not only is IPF more common in regional and remote areas, but that these deaths are distinct and need unique solutions in comparison to IPF occurring in major cities. There is a gap in Australian literature concerning how risk factors of IPF and IPF offenders differ between urban and rural areas, as well as how victims seek help and access services in rural areas. More broadly, international research lacks the addition of Australian data, which would allow for a better understanding of the dynamics seen in the United States and Canada, and if they are reflected in rural IPF in Australia. This research aims to address this gap by exploring IPF in rural areas of Queensland and examining how these cases differ to urban IPF.

## Method

### Data

The Queensland DFVDRU currently holds the most comprehensive data regarding IPF in Queensland, with case files relating to all domestic homicides that have occurred in Queensland since 2006. The DFVDRU examines and analyses the coronial and police investigation of each domestic homicide and all of the relevant data and evidence pertaining to these investigations. Between 2020 and 2021, data collection was undertaken at the Coroners Court where the DFVDRU is located. From the available data, 100 finalized and closed cases of IPF perpetrated from 2006 to 2019 were relevant for inclusion based on the following criteria: perpetrated by a male offender, toward a female victim that they either currently were in an intimate relationship with or had previously been in an intimate relationship. This population size of 100 reflects the extent of IPF in Queensland within this time frame.

### The Definition of Rural in Australia

Australia does not have a standard definition of what denotes an urban area or a regional, rural, or remote one (Campo & Tayton, 2015). The Australian Bureau of Statistics (ABS) uses the Australian Statistical Geographical Standard which accounts for population size, distance to an urban center, and access to goods and services. Other definitions are classified based on solely distance or population size (see Roufeil & Battye, 2008); however, ABS classifications are both the most accessible and are used by the QLD DFVDRU and therefore are used for this research. The ABS’ Personal Safety Survey (2013) found that 29% of Australians reside outside of urban areas, with only 2% living in remote or very remote locations. Over two-thirds of Australia's population lives in major cities and compared to other urbanized countries, Australia has one of the lowest population densities outside of major cities (Baxter et al., 2011). This figure changes drastically when examining Indigenous Australians who make up only 2.4% of Australia's population however make up 15% of those living in remote areas and 49% in very remote areas (Baxter et al., 2011).

### Rural IPF Offender Characteristics

Of the entire population, 53% (*N*  =  53) of the IPFs took place in inner regional, outer regional, remote, or very remote locations, while the remaining 47% *(N*  *=*  *47)* occurred in urban locations. Of the 53 rural cases, the majority occurred in inner (*n*  =  22) and outer (*n*  =  21) regional areas. The remaining 10 cases were located in remote and very remote locations. When examining the demographics of offenders in rural areas, the majority are represented in age groups of 55 or below. Inner and outer regional offenders were mostly represented in age groups below 46 however were present at all age groups between 18 and 25 to 76 and above. Remote and very remote offenders were less spread among the ages, with the three remote offenders between ages 46 and 75, and the seven very remote offenders between the ages of 26 and 55. When examining offender's ethnicity, rural offenders were majority non-Indigenous Australian, followed by Aboriginal and/or Torres Strait Islander. For each type of rural location (inner/outer regional and remote/very remote), there were a number of offenders whose ethnicities could not be determined by the data provided, as can be seen in Table 1.

**Table 1.** Frequency Table of Demographics for Rural IPF Offenders *(N*  *=*  *53)*.

	Inner regional		Outer regional		Remote		Very remote	
	*n*	*%*	*n*	*%*	*n*	*%*	*n*	*%*
Offender age								
18–25	1	4.5	1	4.8	0	0	0	0
26–35	4	18.2	8	38.1	0	0	1	14.3
36–45	8	36.4	4	19	0	0	5	71.4
46–55	5	22.7	5	23.8	2	66.7	1	14.3
56–65	1	4.5	1	4.8	0	0	0	0
66–75	1	4.5	1	4.8	1	33.3	0	0
76 +	2	9.1	0	0	0	0	0	0
Unknown	0	0	1	4.8	0	0	0	0
Offender ethnicity								
Non-Indigenous Australian	10	45.5	7	33.3	1	33.3	1	14.3
Aboriginal/Torres Strait Islander	3	13.6	5	23.8	1	33.3	3	42.9
UK/European	1	4.5	2	9.5	0	0	0	0
New Zealand	1	4.5	0	0	0	0	0	0
Asian	0	0	1	4.8	0	0	0	0
Other	0	0	0	0	0	0	0	0
Unknown	7	31.8	6	28.6	1	33.3	3	42.9
Total	22	100	21	100	3	100	7	100

### Procedure

Each case file was coded based on the presence of the variables listed in Table 2. Case files varied in length and content; however, each had a paper file that contained the coronial findings, including a summary of the basic facts of the case. Most information for coding was taken from paper copies of the coronial investigation documentation and the brief of evidence from the police investigation. Additional information was provided digitally, including audio and video witness and offender interviews, as well as phone records and text messages. Variables were input into an Excel spreadsheet and coded based on the definitions provided in Table 2. All coded data was deidentified before analysis.

**Table 2.** Description of Variables Used.

Variable	Definition
Previous IPV	The offender's history of IPV perpetrated against a previous intimate partner
Criminal history^1^	The offender's history of previous perpetration and indictment of a criminal act
Substance use	The offender's current* misuse of alcohol and other drugs
Mental illness	The offender's current* symptoms or diagnosis that reflects a mental illness
Employment	The offender's current* status of employment
Status of relationship	The current* status of the relationship between the offender and the victim
Coercive control	Offender's behavior that coerces or controls the victim using isolation, entrapment, intimidation, and degradation (see Stark, 2007; Stark & Hester, 2019)
Fear of the offender	The victim's explicit fear of the offender for herself or others
Physical abuse	Abuse that is perpetrated physically, including non-lethal strangulation and sexual abuse
Verbal abuse	Abuse that is spoken or written down
Technology-facilitated abuse	Abuse that is perpetrated through technology, including phone calls, text messages, social networking, and emails
Children living with victim	Children who currently reside with the victim for most or all of the time
Contact with police services	The victim and/or offender accessed policing services, including for temporary protection orders or domestic violence orders^^^
Contact with health services	The victim and/or offender accessed health services, including general practitioners, paramedics, hospital staff, and psychiatrists^^^
Contact with other services	The victim and/or offender accessed other services, including domestic violence services, counseling, and drug therapy^^^
Concealment	The offender attempted to conceal the IPF using tactics including disposing of evidence, misleading police, and family and friends
Planning	The offender planned the IPF by purchasing weapons/restraints, met with the victim with the intent to commit IPF
Cause of death	The cause of death ruled by the relevant coroner in each case
Homicide suicide	The offender successfully or attempted to commit suicide immediately after the IPF
CALD	The victim is from a culturally or linguistically diverse background

*Current at the time of the IPF.

^ When relevant to the relationship between the victim and offender.

Identification of these variables at times relied on the offender or those connected to the victim and offender disclosing information such as substance misuse, mental illness, or if the victim feared the offender. Official records included in the coronial files, such as medical, employment, corrections, or police records were also used to determine the presence of these variables in each case. It is noted that in cases where mental illness was solely used as a defense tactic in legal proceedings, this factor was not identified as present. Due to the limited number of cases in Queensland, specific categories of mental illness were excluded in reporting. This ensured identification of offenders could not occur and was further informed by the harms of the perpetuation of the myth that mental illness and violence are linked, which causes the stigmatization of certain conditions (Pescosolido et al., 2019). It was determined that as this study's focus is not the role of mental health in offending, it was appropriate to exclude this information from the dataset.

Risk factors such as coercive control, physical abuse, verbal abuse, and technology-facilitated abuse are difficult to define and measure (Dragiewicz et al., 2018; Harris & Woodlock, 2021; Stark & Hester, 2019) particularly posthumously as victims are unable to report on the violence experienced and offenders are likely to minimize and deny perpetration (Bancroft, 2003; Dekeseredy, 2011). As such, it is likely that these forms of violence are underrepresented in the data, and this is noted as a limitation of posthumous data collection. These risk factors may also overlap, coercive control involves physical, verbal, and technology-facilitated abuse; however, in order to capture the fullest extent of forms of violence, the methodological decision was made to separate variables where information was consistently identifiable and combine variables in which data consistently lacked sufficient detail (for example specific physical manifestations of violence such as sexual violence and non-lethal strangulation).

### Analyses

Deidentified data were input into Statistical Package for the Social Sciences (SPSS; Version 27.0) and was cleaned and checked for errors. A frequency analysis was run to obtain offender age and ethnicity demographics for each type of rural area; inner regional, outer regional, remote, and very remote. Then the data were split using a binary variable of rural and urban, with urban including major cities and rural including the other four categories. A binary variable was chosen in this case considering the smaller number of cases in the remote and very remote categories. As the purpose of this research is to examine the differences between urban and rural spaces, it was not necessary to separate variables between the types of rural areas. Frequency analysis was run on the binary variable of location using the variables defined in Table 2 (excluding the variable CALD). Chi-square analysis was conducted on two separate variables, concealment and CALD status. Missing data were included in frequency tables but were excluded from chi-square analysis.

## Results

### Common Risk Factors in Rural IPF

When examining the location of femicide as a binary variable, several comparisons of frequency of risk factors can be made between IPFs that occurred in rural areas and those that occurred in urban areas. When examining the frequency of offender characteristics in both urban and rural cases of IPF, a visual inspection of Table 3 does not highlight any significant differences in offender's use of substances.

**Table 3.** Urban and Rural IPF Offender Characteristics.

	Urban		Rural	
	*n*	*%*	*n*	*%*
Previous IPV				
None	28	59.6	24	45.3
Nonphysical	1	2.1	3	5.7
Physical	0	0	5	9.4
Physical and nonphysical	12	25.5	8	15.1
Unknown	6	12.8	13	24.5
Criminal history				
Yes	24	51.1	29	54.7
No	19	40.4	13	24.5
Unknown	4	8.5	11	20.8
Substance use				
Yes	21	44.7	26	49.1
No	20	42.6	19	35.8
Unknown	6	12.8	8	15.1
Mental illness				
Yes	22	46.8	21	39.6
No	24	51.1	20	37.7
Unknown	1	2.1	12	22.6
Employment				
Employed	27	57.4	18	34
Unemployed	13	27.7	17	32.1
Retired	5	10.6	2	3.8
Unknown	2	4.3	16	30.2
Status of relationship				
Current relationship	21	44.7	29	54.7
Pending/actual separation	26	55.3	23	43.4
Unknown	0	0	1	1.9
Total	47	100	53	100

Variables including an offender's previous DV perpetration, criminal history, presence of mental illness, and employment status do show differences in frequency between locations; however, when accounting for the larger amount of missing data for rural cases, these differences are not statistically significant. About half of all offenders had a criminal history and just under half of all offenders misused substances. The status of relationship at the time of the IPF appears to differ between urban and rural locations with the majority (55.3%) of urban IPFs perpetrated during or after separation while rural IPFs were more likely to occur within a current relationship (54.7%).

The frequency of commonly associated risk factors of IPF appears to be similar between urban and rural locations. Variables in Table 4 such as coercive control, physical abuse, and verbal abuse were in the majority (>55%) of cases in both urban and rural locations. A victim's fear of the offender was less common for both urban (48.9%) and rural (45.3%) cases; however, as shown in the amount of missing data, this variable was difficult to determine posthumously without a report from a corroborating witness or other sources.

**Table 4.** Risk Factors Associated With IPF in Urban and Rural Queensland.

	Urban		Rural	
	*n*	*%*	*n*	*%*
Coercive control				
Yes	35	74.5	32	60.4
No	6	12.8	5	9.4
Unknown	6	12.8	16	30.2
Fear of the offender				
Yes	23	48.9	24	45.3
No	11	23.4	5	9.4
Unknown	13	27.7	24	45.3
Physical abuse				
Yes	26	55.3	30	56.6
None reported	20	42.6	19	35.8
Unknown	1	2.1	4	7.5
Verbal abuse				
Yes	33	70.2	30	56.6
None reported	8	17.1	9	17
Unknown	6	12.8	14	26.4
Technology-facilitated abuse				
Yes	20	42.6	12	22.6
No	15	31.9	18	34
Unknown	12	25.5	23	43.4
Children living with victim				
Yes	17	36.2	17	32.1
Yes—children harmed	1	2.1	4	7.5
Yes—children killed	2	4.3	2	3.8
No	27	57.4	30	56.6
Total	47	100	53	100

The presence of children residing with the victim at the time of the IPF was unlikely in both urban and rural locations with 57.4% of victims in urban locations and 56.6% of victims in rural locations having no children living at their residence at the time of the IPF. Similar to Table 2, any differences in frequencies for variables, for example, technology-facilitated abuse (present in 42.6% of urban cases and 22.6% of rural cases), could be accounted for by the number of unknown factors in the rural cases of IPF (43.4%).

Table 5 shows the level of contact the victim and offender had with services, including health, police, and other services. Overall, both urban and rural victims and offenders had low contact with services.

**Table 5.** Victim and Offenders Contact With Services in Urban and Rural Areas.

	Urban		Rural	
	*n*	*%*	*n*	*%*
Police services				
None	25	53.2	24	45.3
Yes—victim	2	4.3	0	0
Yes—victim and offender	18	38.3	19	35.8
Unknown	2	4.3	10	18.9
Health services				
None	24	51.1	27	50.9
Yes—offender	7	14.9	3	5.7
Yes—victim	3	6.4	6	11.3
Yes—victim and offender	9	19.1	5	9.4
Unknown	4	8.5	12	22.6
Other services				
None	29	61.7	28	52.8
Yes—offender	3	6.4	3	5.7
Yes—victim	4	8.5	6	11.3
Yes—-victim and offender	6	12.8	2	3.8
Unknown	5	10.6	14	26.4
Total	47	100	53	100

Contact with police services was comparable between urban and rural cases, with the largest difference apparent in the way urban victims accessed policing services without the offender making similar contact (4.3%) when compared to rural victims (0%). The frequency of contact with health services was similar between urban and rural cases however there is a larger amount of missing data in rural victim and/or offender contact (22.6%). Overall, other services were accessed the least by victims and/or offenders in both urban (61.7%) and rural settings (52.8%).

As is shown in Table 6, examining the frequency of IPF characteristics and pre- and post-IPF offender behaviors in both rural and urban environments shows some differences.

**Table 6.** IPF Characteristics and Pre- and Post-IPF Offender Behaviors in Urban and Rural Areas.

	Urban		Rural	
	*n*	*%*	*n*	*%*
Concealment*				
Yes	25	53.2	18	34
No	21	44.7	34	64.2
Unknown	1	2.1	1	1.9
Planning				
Yes	20	42.6	16	30.2
No	26	55.3	35	66
Unknown	4	8.5	2	3.8
Cause of death				
Wound from sharp object	17	36.2	22	41.5
Strangulation/suffocation	9	19.1	2	3.8
Gunshot wound	4	8.5	10	18.9
Blunt force trauma	4	8.5	9	17
Other	5	10.6	2	3.8
Unascertained	8	17	8	15.1
Homicide suicide				
Yes	7	14.9	13	24.5
Attempted	8	17	7	13.2
No	32	68.1	33	62.3
Total	47	100	53	100

*Pearson chi-square test.

Offenders who concealed the IPF were more likely to be in an urban area (53.2%) than in a rural area (34%). Urban offenders were also slightly more likely to have evidenced planning of the IPF (42.6%) than rural offenders (30.2%). Both urban (36.2%) and rural (41.5%) IPF victims were most likely to have died from a wound from a sharp object; however, it was more likely for a rural IPF victim to have died from a gunshot wound (18.9%) than an urban IPF victim (8.5%). Urban IPF victims’ rate of strangulation or suffocation was much higher (19.1%) than rural IPF victims (3.8%). The difference in frequency of the offender suicide after the IPF was small with rural offenders slightly more likely to successfully suicide (24.5%) than urban offenders (14.9%); however, when considering suicide attempts, urban offenders account for slightly more (17%) attempts than rural offenders (13.2%)

### The Link Between Location and IPF Risk Factors

To examine the relationship between rural living and the IPF risk factors that exist in the data, chi-square tests were determined to be most suitable for the collected variables. These tests were performed on the binary variable of location, residing in a rural location or residing in an urban location. Of the available risk factors, two were found to be related at a significant level. For each chi-square test, a crosstabulation was run to determine initial relationships and to ascertain if any assumptions would be violated using the available data.

A chi-square test of independence was performed to examine the relation between offenders living in a rural area and their concealment of the IPF. The relationship between these variables was significant, *X^2^* (1, *N*  =  98)  =  3.9, *p*  =  .049. This suggests there is a significant relationship between residing in a rural area and concealment. The effect size for this finding, *Phi,* was moderate, −.198 (Cohen, 1988) and demonstrates a negative association meaning that concealment behaviors are less likely to be undertaken by rural offenders.

Another chi-square test of independence was performed to determine the significance of the relationship between living rurally and a victim's status of being CALD. The relationship between these variables was significant, *X^2^* (1, *N*  =  100)  =  6.5, *p*  =  .011. The effect size, *Phi*, was moderate, −.255 (Cohen, 1988) and indicates that living rurally is negatively associated with being CALD.

## Discussion

While the existing literature concerning IPV and IPF in rural areas suggests key differences in certain risk factors when compared to urban spaces, there are no Australian studies that have examined these differences in rural Queensland or anywhere else in the country. Within a period of 14 years, the majority (53%) of IPFs occurred in rural areas of Queensland with 22% in inner regional locations, 21% in outer regional locations, 3% in remote locations, and 7% in very remote locations. While this overall rate aligns with international findings, there are differences in how this IPF presented in these areas when compared to other studies.

### IPF Perpetrators in Rural Spaces

Overall, IPF in rural areas is similar to those that occurred in urban areas on a number of key features. When examining the risk factors that were common in offenders residing in rural spaces compared to those in urban ones, most factors did not differ significantly between the two. Previous research has suggested differences between urban and rural offenders; however, these results highlight the similarities of IPF offenders across both environments. Criminal history, substance use, employment status, and mental illness were not impacted by the location of the IPF. Offender's history of DV with a previous partner did show some differences across spaces, with those in rural areas being the only offenders to have perpetrated only physical abuse toward a previous partner, differing from those who perpetrated both physical and nonphysical abuse in urban cases. While both spaces most frequently had offenders who were not known to have committed any previous DV, the unique result in rural areas suggests that rural offenders may be more physically violent in their previous DV, or that nonphysical abuse has not been appropriately captured in these offenders previously. IPF offenders did differ between urban and rural areas when examining the status of the relationship at the time of the IPF. In urban areas, offenders were more likely to be separated or pending separation from the victim, while in rural areas, offenders were more likely to be in a current relationship. This suggests that the traditional values of rural areas discourage separation, even in cases of potential abuse, or that victims are hesitant to attempt to separate from offenders due to the physical isolation of rural spaces.

### IPF Risk Factors in Rural Spaces

The common risk factors among the population of both urban and rural IPFs included coercive control, verbal abuse, physical abuse, technology-facilitated abuse, a victim's fear of the offender, and the presence of children living with the victim. There were no significant differences between locations for these risk factors which suggests that IPF in Queensland may have the same risk factors in both rural and urban spaces and differs more on the severity and length of victimization that other international studies have reported.

### How IPF Offenders and Victims Access Services

Research has consistently found that access to services and formal supports is challenged by rural spaces. Literature examining IPV victim/survivors in Australia and internationally suggests that those living in rural areas face more barriers in accessing police, medical aid, and DV services. The results of this research however show that rates of contact with services including police, health, and DV services did not differ in location as expected and instead remained low between victims and offenders for both urban and rural areas. The number of unknown values is also notable when examining the rate of contact with services in rural areas as this value is much higher than in urban areas across all three services; police, health, and DV related. This suggests a lack of comprehensive data gathered and stored on rural IPF, or a lack of reporting from rural services overall.

### IPF Characteristics and Offender Behaviors in Rural Spaces

Examining the differences between the IPF characteristics and pre- and post-IPF behaviors in urban and rural spaces, the presence of planning prior to the IPF and the rate of homicide suicide did not change significantly. Planning has been identified more recently by scholars as a characteristic of IPF overall (Ferguson, 2019; Monckton Smith, 2020). Monckton-Smith proposes planning as the seventh stage of the timeline intimate partner homicide offenders follows, the last stage before the homicide. While this factor was present in just over a third of the cases (36%), it is noted that these cases align with Monckton Smith's (2020) eighth-stage homicide timeline. For cause of death, it appears that gunshot wounds were more likely to be the cause of death in rural areas than in urban ones and this was the second highest cause of death rurally. This is expected considering research that highlights firearm ownership as a risk factor for IPF and the higher rate of firearm use and ownership in rural areas in Australia (Harris, 2016; Spencer & Stith, 2020). Concealment behaviors post-IPF were also much less common in rural offenders when compared to urban ones. This result indicates that perpetrators are using the natural isolation of the environment as an alternative to concealment or that IPF offenders in rural areas are less concerned with detection avoidance than urban offenders who are more likely to undertake concealment behaviors than not. This is further corroborated by the test of independence conducted that highlighted the negative association between rural areas and concealing behaviors.

### CALD Women in Rural Spaces

Much research conducted nationally suggests that rural areas are often most populated with white, anglo-centric persons who would identify as Australian. The results in this study confirm this and show a negative association between a victim's status of being CALD and living in a rural area. While being CALD in these areas is a risk factor for IPV due to the increased barriers these women face (Campo & Tayton, 2015), the lack of CALD victims in this population likely reflects the lack of CALD women living in rural Queensland overall.

### Limitations

While results of common risk factors in rural IPF perpetrators generally align with the wider research available, there are some limitations to this study that need to be discussed. Although there is some research concerning IPF in rural areas, the majority of this research has been conducted in countries other than Australia such as the U.S. and Canada. As a result, the comparisons drawn between this research and existing studies are limited, although remain valuable for furthering the understanding of IPF offenders on an international scale. The United States does have similarities in presentations of IPF and risk factors; however, the composition of the country differs from the large areas of rural and remote land in Australia (Hugo, 2002). Canada holds some similarities in the extent of rural and isolated spaces and as such, the Canadian landscape allows for a more fitting comparison.

Following from this, because this study did not separate rural locations into the specific categories of inner regional, outer regional, remote, and very remote, some of the nuance to these data is lost. Due to the small population size, this methodological decision was made for ease of interpretation and was found to suit the purpose of this study which was to compare urban and rural areas in Queensland. Further due to the population size and low homicide rate, many of the statistical analyses run could not be determined to be significant. It should be noted that all finalized and closed Queensland IPF cases from 2006 were examined and IPF prior to 2006 does not have comprehensive data available for study. The limited Australian research of rural IPFs and the low homicide rate calls for this study to be replicated on a wider, national scale to access a larger population and sample size and add to the body of literature on this topic.

Lastly, as can be seen in the above tables, much of the data concerning rural IPFs are missing or incomplete. Because of this, some risk factors cannot be reported on and a limited number could be inaccurate; however, this does not undermine the results. This lack of data has improved over time, with earlier data less complete than later data. More comprehensive case files were created with the development of the QLD DFVDRU; however, in light of the rates of IPF in rural areas and the increasing move toward relocation to rural areas in a response to COVID-19, it is highlighted that more in-depth record-keeping and investigation must be undertaken in regional and remote locations of Queensland. It is noted that the data may further be an underrepresentation of the true extent of violence perpetrated by offenders due to the difficulty in capturing data posthumously. This is a limitation of utilizing any data gathered after the IPF has occurred and highlights the rate of more covert risk factors such as coercive control, verbal abuse, and technology-facilitated abuse is likely higher than reported.

### Implications for Future Study and Responses

Policies and strategies implemented to prevent homicide are often designed with urban areas in mind and are therefore more useful to those living in cities as they align with the cultural and geographic norms of metropolitan life (Gallup-Black, 2005). This highlights the current urban-centric bias of DV policies and services that currently exist for victim/survivors. While those in rural areas and those who are not able to financially support themselves are highlighted as vulnerable, the barriers they face to access services are not considered within the services currently available (Owen & Carrington, 2015). Service providers in rural areas are aware of these issues and attempt to provide tailored assistance in these cases; however, more specific policies need to be implemented for victim/survivors, particularly for cases of IPV that have risk factors for IPF. In George and Harris (2014) study, they recommend targeted education for rural community sectors, specialized training for police, and more funding and resources directed toward increasing and maintaining DV services in rural areas. These recommendations align with the current research, as the lack of consistent data throughout the variables suggests a lack of reporting, a lack of community understanding and awareness, and a lack of validation from rural policing and health services.

Due to the low contact with services across both urban and rural spaces highlighted in this research, other responses should also be considered. Straatman et al. (2020) suggest implementing home visits in rural areas could be effective in providing ongoing support while developing relationships with victim/survivors. This could address issues with victim/survivors being ‘outed’ in public places using DV services. Because of the increased rate of gunshot wounds as a cause of death for rural victims, firearm ownership and use should be addressed. Doherty and Hornosty (2008) suggest that gun safety commercials and pro-removal firearm policies should be targeted toward rural communities. They recommend, in known cases of IPV, to impose firearm restrictions on offenders and that victim/survivors should be further encouraged to consider personal safety and risk when firearms are present.

The findings of this study are unique and are the first to highlight and explore the rate of IPF in rural areas of Queensland, Australia. Common risk factors for IPF and help-seeking behaviors appear to be similar across urban and rural spaces, suggesting that despite international findings, the environment in which the violence occurs does not shift the type of abuse or how the victim understands their risk of harm in Australia. The results show that while IPF in rural areas presents as similar to urban areas, the vastness of rural spaces and the associated physical isolation lends itself to increased rates of IPF. Dangerous relationships are sustained by victims, suggesting the barriers to escape their abuser lie in the physical environment of rural areas. Further, offenders are less likely to conceal their behaviors related to the IPF, indicating the isolated nature of rural areas may remove the need for detection avoidance or concealment. Offenders do not differ in their characteristics, and this could imply that the type of offender does not impact rates of IPF when examining the spaces in which they occur.

These findings are key in understanding the increased rate of IPF in rural Queensland as well as in furthering the international examination of IPF offenders and the characteristics they share or are separated by. Future studies should further examine offender behavior and characteristics between urban and rural spaces and the impact this has on risk factors of IPF. Finally, more research is needed on how the social and physical isolation of rural areas contributes to help-seeking behaviors of victims and offenders in both urban and rural spaces.

## Notes

1. It is noted that the definition of criminal history was purposefully inclusive of a broad range of criminality as data was often sporadic in reference to specific historical criminal acts. General offending has been cited as a good predictor of violent recidivism, including IPF, and as such this definition was found to be an appropriate measure for the study (see Bridger et al., 2017).
